# Gait and Foot Clearance Parameters Obtained Using Shoe-Worn Inertial Sensors in a Large-Population Sample of Older Adults

**DOI:** 10.3390/s140100443

**Published:** 2013-12-27

**Authors:** Farzin Dadashi, Benoit Mariani, Stephane Rochat, Christophe J. Büla, Brigitte Santos-Eggimann, Kamiar Aminian

**Affiliations:** 1 Laboratory of Movement Analysis and Measurement, École Polytechnique Fédérale de Lausanne (EPFL), Lausanne 1015, Switzerland; E-Mail: benoit.mariani@epfl.ch; 2 Service of Geriatric Medicine and Geriatric Rehabilitation, University of Lausanne Medical Center, Epalinges 1066, Switzerland; E-Mails: stephane.rochat@chuv.ch (S.R.); christophe.bula@chuv.ch (C.J.B.); 3 Institute of Social and Preventive Medicine (IUMSP), University of Lausanne Medical Center, Lausanne 1010, Switzerland; E-Mail: brigitte.santos-eggimann@chuv.ch

**Keywords:** gait, elderly, foot clearance, ambulatory system, sensor fusion, reference data

## Abstract

In order to distinguish dysfunctional gait, clinicians require a measure of reference gait parameters for each population. This study provided normative values for widely used parameters in more than 1,400 able-bodied adults over the age of 65. We also measured the foot clearance parameters (*i.e.*, height of the foot above ground during swing phase) that are crucial to understand the complex relationship between gait and falls as well as obstacle negotiation strategies. We used a shoe-worn inertial sensor on each foot and previously validated algorithms to extract the gait parameters during 20 m walking trials in a corridor at a self-selected pace. We investigated the difference of the gait parameters between male and female participants by considering the effect of age and height factors. Besides; we examined the inter-relation of the clearance parameters with the gait speed. The sample size and breadth of gait parameters provided in this study offer a unique reference resource for the researchers.

## 1. Introduction

The aging population requires mobility related therapeutic and/or rehabilitative care that concerns a substantial part of resources in every healthcare system. Motion capture system is a key component of modern therapeutic and rehabilitative programs [1]. Although a significant part of our current understanding of human locomotion is owing to the use of optical motion capture systems, these systems are generally restricted to in-lab conditions. Suffice it to say that the measurement in laboratory can impose conditions that are significantly different than that of free daily ambulation.

The advances in miniaturized body-worn measurement systems enabled a long-term recording of kinematics during both in-lab and daily life activities [2,3]. Currently, these systems made motion detection feasible by using inertial sensors, in combination with magnetic and/or force sensors [4]. In addition to a measurement system, appropriate algorithmic approaches are needed to accurately delineate limb's trajectory and extract clinically relevant parameters e.g., as in a wearable gait analysis system [5]. The extraction of trajectory using body fixed sensor relies on a 2D or 3D kinematic model that takes into account the limb's workspace.

The foot trajectory tracking can be used for a comprehensive study of fall in old age [6]. Fall is considered to be a major source of morbidity and mortality in older adults and imposes huge costs to the healthcare systems [7]. The classical foot trajectory descriptors such as stride length, stride velocity and temporal parameters have been extensively investigated to determine the fall related factors [5,8,9]. When the swing foot progression is unexpectedly obstructed, a trip occurs that leads to a forward rotation of the body and eventually might cause a fall. About 53% of falls happen due to tripping [10,11], which indicates the importance of the swing foot trajectory scrutiny. Nevertheless, clinical implications of foot clearance parameters amongst old population and their inter-relation with other gait parameters have not been adequately explored. The mean and SD values of clearance parameters reported for different age groups were not consistent in the literature [12–14] since small populations were studied. This small sample size is a natural consequence of complexity of measurement in gait laboratories. Moreover, assessment of gait variability based on limited field of view of camera-based motion capture systems (and thereof limited number of cycles) can be misleading.

The inertial measurement unit (IMU) has been employed to estimate just a limited subset of foot clearance parameters [5,15]. On the other hand, by employing the IMU the measurement protocol is not anymore restricted to the in-lab capture volume. Besides, a continuous recording of the motion signals is possible contrary to the standard optical motion capture techniques when occlusion of markers could lead to loss of a part of movement trajectory.

In view of the introduced problems, this study proposes the application of a shoe-worn IMU to investigate several foot clearance parameters as well as other gait parameters in a clinically relevant setting. We employed the method introduced by Mariani and co-workers in [6] to extract these parameters from gait kinematics on a population-based cohort of community-dwelling 66 to 77 year old individuals. In the second part of this paper we summarized the algorithmic approach to extract the gait temporal, spatial and clearance parameters. The third part of the study has two main focuses. Primarily, we report the normative values of gait spatiotemporal as well as foot clearance parameters based on age group and gender that can be used as a reference for clinical research. Next, we demonstrated the inter-relation of clearance parameters with gait speed that is the hallmark of gait performance assessment in older population. This latter study helps to better realize the significance of clearance parameters as fall predictors in older persons.

## 2. Experimental Section

### 2.1. Shoe-Worn IMU for Data Acquisition and Calibration

**Figure 1.**
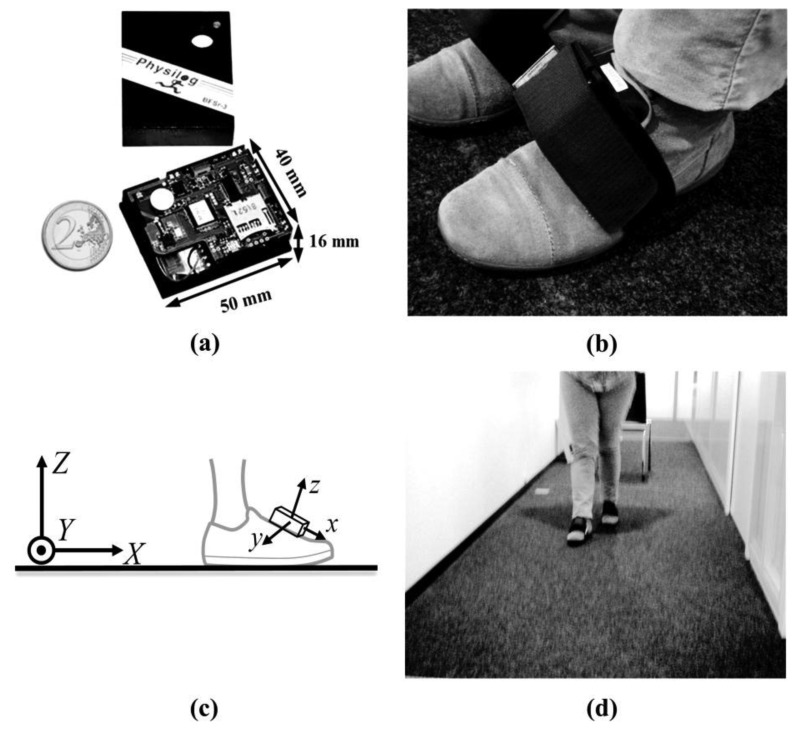
(**a**) A wireless Physilog^®^ IMU; (**b**) IMU attachment to the shoe with the elastic strap; (**c**) Illustration of the orientation of the IMU relative to the global frame of the measurement; (**d**) An example of the 20 m walking trial in the corridor.

Two Physilog^®^ (Gait Up, Lausanne, Switzerland) were used in this study. Physilog^®^ is an IMU based is a standalone device (dimensions: 50 mm × 40 mm × 16 mm, weight: 36 g) including a tri-axial accelerometer (MMA7341LT, range ±3 g, Freescale, Austin, TX, USA), a tri-axial gyroscope (ADXRS, range ±600 °/s, Analog Devices, Norwood, MA, USA), a battery (3.7 V, 595 mAh), a memory unit and a microcontroller (Figure 1a).

The kinematics data (3D acceleration and 3D angular velocity) were sampled on 16 bits at a frequency of 200 Hz and then low-pass filtered at 17 Hz [16] and recorded on the μSD card before transferring to the PC. Signals from two Physilog^®^ sensors were synchronized wirelessly. The sensor can be easily fixed on the upper part of the shoe with an elastic strap as shown in Figure 1b. Shape memory foam beneath the sensor is used to guarantee comfort and stable positioning of the system.

In order to be sure that the measurement was not affected by the sensor location on the foot, each IMU frame was aligned with the foot walking frame during each walking trial according to [5]. In the first step by assuming that the pitch angular velocity is maximal in the sagittal plane, the IMU's *y*-axis was aligned to the principal axis of the measured angular velocity (*Y*) (see Figure 1c). Then, in the absence of foot movement during foot-flat the sensor inclination measured by accelerometer was set to null in order to align *z*-axis to *Z*. The third aligned axis (*x*-axis) has been accordingly determined as the cross product of the two other aligned axes.

### 2.2. Measurement Protocol

**Figure 2.**
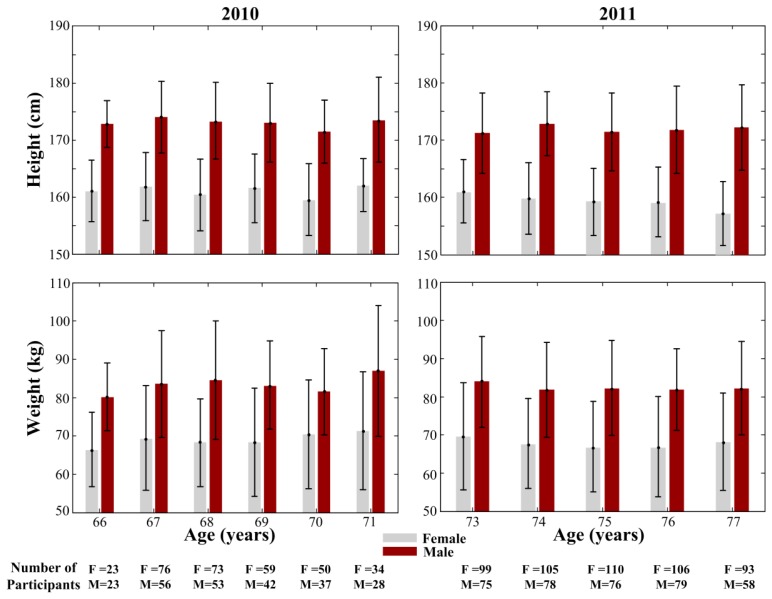
Demographics of the participants in 2010 (*n* = 554) and 2011 (*n* = 879) studies.

The Lc65+ study includes two representative samples of the community-dwelling population of Lausanne city enrolled at the age of 65 to 70 in 2004 and 2009. Anthropometric measurements and walking tests are performed in the study center by trained medical assistants first during the year following enrollment (initial) and then during triennial examinations (follow-up). Physilog^®^ recording of gait parameters was introduced in 2010, after a familiarization session for medical assistants, in the course of the initial assessment of the sample enrolled in 2009 (aged 66 to 71); of 1,245 participants, only those assessed between June 18 and December 15, 2010 used Physilog (*n* = 554, 44.5%). This sample may be slightly biased due to the postponement of some appointments to the second half of the year in case of hospitalization or active illness. Physilog^®^ was also used in 2011 for the whole sample enrolled in 2004 (aged 73 to 78): recordings were obtained for 879 of 963 (91.3%) subjects who attended the follow-up assessment at the study center. We therefore report the spatiotemporal and clearance parameters of 2010 and 2011 studies separately in this article. The gait parameters were extracted during a 20 m walking trial in a corridor at a self-selected pace as demonstrated in Figure 1d. A continuous monitoring of the quality of Physilog records ensured a correct use of the device by medical assistants. Figure 2 shows the demographic information of the participants who used Physilog^®^.

### 2.3. Estimation of Gait Descriptors

Gait spatiotemporal descriptors and foot clearance parameters were estimated from IMU 6D signals using methods proposed in [5,6,17,18]. The parameters extraction procedure is briefly explained in the following paragraphs.

#### 2.3.1. Estimation of Stance Temporal Phases

The stance phase is the period between initial contact, referred to as Heel-Strike (HS), and terminal contact, referred as Toe-Off (TO). The instant when toes touch the ground during stance, is referred as Toe-Strike (TS), and the instant when the heel rises from the ground, is called Heel-Off (HO). Accordingly, HS, TS, HO, TO are considered as the temporal events of stance. Rule-based event detection on the foot kinematic signals, was used to extract these temporal events [17].

The detected temporal events at each cycle were then used to quantify the stance and inner-stance phases. The stance period at cycle *k*, thus can be calculated as:

(1) $$\Delta T_{St}^{k}=t(TO^{k})-t(HS^{k})$$

where *t*(. ) denotes the instant when the temporal event occurred. Similarly, the duration of the three inner-stance phases, including loading response (*Load*), foot-flat (*ff*) and push-off (*Push*), have been determined by Equations (2)–(4):

(2) $$\Delta T_{\text{Load}}^{k}=t(TS^{k})-t(HS^{k})$$

(3) $$\Delta T_{ff}^{k}=t(HO^{k})-t(TS^{k})$$

(4) $$\Delta T_{\text{push}}^{k}=t(TO^{k})-t(HO^{k})$$

Considering *N_S_* as the number of steps at each measurement, the gait cycle duration ( $\Delta T_{\text{Cyc}}^{k}$) and the cadence were also extracted as two other temporal parameters:

(5) $$\Delta T_{\text{Cyc}}^{k}=t(HS^{k})-t(HS^{k-1})$$

(6) $$\text{Cadence}=60N_{s}/\sum_{k=1}^{N_{S}}\Delta T_{\text{Cyc}}^{k}$$

#### 2.3.2. Estimation of Spatial Gait Descriptors

At instant *i* = *1*,*2*,…,*N* of cycle *k*, the orientation of the foot relative to the global frame *R^k^*(*i*) was calculated by strap-down integration of the angular velocity vector [5]. The initial orientation *R^k^*(0) was obtained by using the acceleration signal during motionless period of $\Delta T_{ff}^{k}f$. Subsequently, the gravity-free acceleration in the global frame (*a⃗_GF_*) can be calculated by Equation (7):

(7) $${\vec{a}}_{GF}^{k}(i)={\vec{a}}^{k}(i).R^{k}(i)-\vec{g},\vec{g}=[0,0,1]$$

Foot velocity ( ${\vec{v}}_{GF}^{k}$) and position ( ${\vec{p}}_{GF}^{k}$) were estimated from the numerical integration of (*a⃗_GF_*) and drift removal technique using the zero velocity update during the foot-flat period [5].

**Figure 3.**
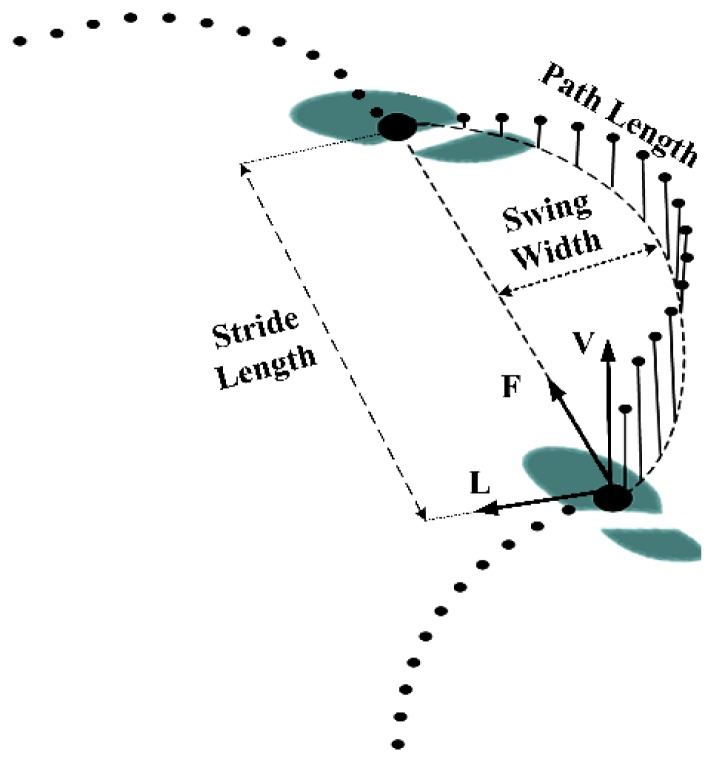
Illustration of the frontal-lateral-vertical (FLV) walking frame and four classic spatial gait parameters.

At each cycle four parameters have been extracted as the spatial gait descriptors of the foot trajectory (Figure 3). The first parameter is the stride velocity (SV) that is the average of ${\vec{v}}_{GF}^{K}$projection in the horizontal plane of walking during two successive foot-flats. By representing the ${\vec{p}}_{GF}^{k}$

in the frontal-lateral-vertical (FLV) walking frame, the stride length (SL) can be defined as the linear distance between two successive foot-flat positions in frontal axis [18]. The swing width (SW) was then defined as the maximum lateral deviation of the foot trajectory during the swing phase. The path length (PL) is defined as the length of 3D curve ${\vec{p}}_{GF}^{k}$ normalized by the stride length.

#### 2.3.3. Estimation of Heel and Toe Clearance Parameters

The clearance parameters represent the extremes of the heel and toe trajectory during the swing phase. The 2D kinematic model proposed by Mariani and co-workers [6] has been adopted to estimate the IMU position relative to the foot in order to calculate the heel and toe trajectories. Suppose *θ_Y_* be the pitch angle at heel strike after applying linear drift compensation between two successive foot-flat periods [19]. Moreover, suppose $Z_{GF}^{k}(i)$ the vertical component of ${\vec{p}}_{GF}^{k}$ from Section 2.3.2 at instant *i* of the *k^th^* cycle.

**Figure 4.**
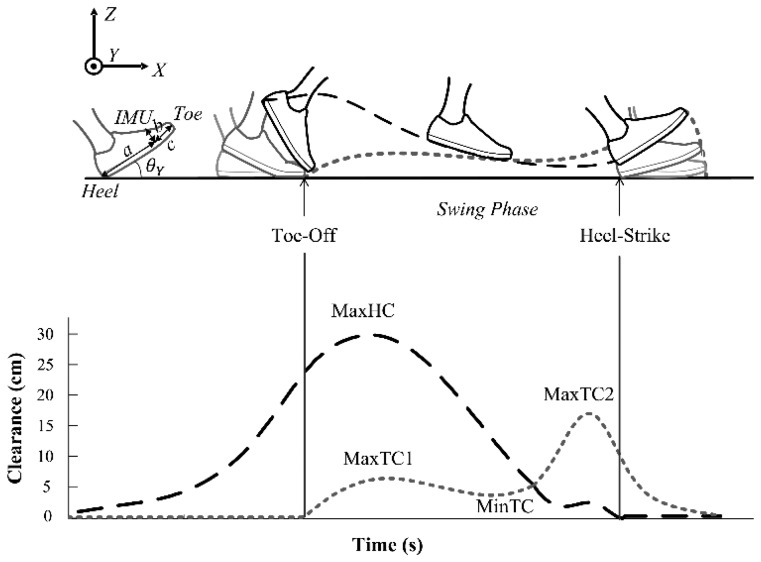
Illustration of the heel (black line) and toe (light gray line) trajectories during swing phase and corresponding clearance parameters. IMU location relative to the heel and toe can be determined with the three parameters *a*, *b*, *c*. Besides, *θ_Y_* shows the heel pitch angle during the stance phase.

By defining the IMU distance with regard to the heel and toe as three unknowns *a*, *b*, *c* as depicted in Figure 4, the vertical trajectory of the IMU ( $Z_{\text{IMU}}^{k}$), heel ( $Z_{\text{Heel}}^{k}$) and toe ( $Z_{\text{Toe}}^{k}$) are:

(8) $$Z_{\text{IMU}}^{k}(i)=Z_{GF}^{k}(i)+b$$

(9) $$Z_{\text{Toe}}^{k}(i)=Z_{GF}^{k}(i)-b.\text{cos}\left(\theta_{Y}(t)\right)+c.\text{sin}\left(\theta_{Y}(t)\right)$$

(10) $$Z_{\text{Heel}}^{k}(i)=Z_{GF}^{k}(i)-b.\text{cos}\left(\theta_{Y}(t)\right)-a.\text{sin}\left(\theta_{Y}(t)\right)$$

In line with [6], we used the constraints in Equation (11) to estimate *a*, *b* and *c* and subsequently toe and heel trajectories:

(11) $$\{\begin{array}{c} Z_{\text{Toe}}^{k}(TO^{K})=0\\ Z_{\text{Heel}}^{k}(HS^{k})=0\\ a+c=\text{ShoeSize} \end{array}$$

Considering all cycles, a least square solution was used to estimate *a*, *b* and *c* and the following ground contact constraints were considered in order to correct the 2D trajectory of the toe and heel (Figure 4):

(12) $$\{\begin{array}{c} \forall t\in \left[T_{ff}^{k},TO^{k}\right]:Z_{\text{Toe}}^{k}(t)=0\\ \forall t\in \left[HS^{k},T_{ff}^{k}\right]:Z_{\text{Heel}}^{k}(t)=0 \end{array}$$

The corrections in Equation (12) do not lead to discontinuities in the toe and heel trajectories since the equations have been solved with null toe and heel elevation at *TO^k^* and *HS^k^* instants.

Six clearance parameters are extracted to represent the foot clearance. The first parameter is the heel strike pitch angle (*θ_Y_*). Moreover, at each cycle maximum of the heel clearance (MaxHC), the first and second local maxima of the toe clearance (MaxTC1 and MaxTC2 respectively) and minimum of the toe clearance (MinTC) have been extracted from the clearance trajectories. The last parameter is the velocity of foot at minimum of the toe clearance (V_MinTC_).

### 2.4. Data Analysis

The parameters were classified into three sets *i.e.*, temporal ( $\Delta T_{\text{Cyc}}^{k}$, $\Delta T_{St}^{k}$, $\Delta T_{\text{Load}}^{k}$, $\Delta T_{ff}^{k}$, $\Delta T_{\text{Push}}^{k}$, Cadence), spatial (*SV*, *SL*, *SW*, *PL*) and clearance parameters (*θ_Y_*, MaxHC, MaxTC1, MaxTC2, MinTC, V_MinTC_). For each participant the extracted parameters were represented by the average and standard deviation over all the cycles of the measurement session. The effects of gender and age on the three sets of parameters were analyzed with analysis of variance (ANOVA). Next, the average, standard deviation, median, 10th and 90th percentiles over all participants were calculated (based on individual average and standard deviation of the parameters). We used the Wilcoxon rank-sum test to detect the existence of a significant difference between the medians of the parameter in women and men (significance level *p* < 0.05). The Pearson correlation between temporal and clearance parameters as well as between spatial and clearance parameters was investigated.

## 3. Results and Discussion

Although previous studies have presented typical values for gait spatiotemporal parameters [20,21], our work provides a more inclusive dataset both in terms of number of participants and also number of studied parameters. To our knowledge, the present study is the first one that includes normative reference values on clearance parameters. The 2010 data analysis involves 544 participants and 2011 includes the data of 879 participants. During the measurements in this study, the participants wore the IMUs on both shoes. Subsequently, the result of measurement is available for both sides. However, for the sake of conciseness we just report the result on the right side. For each participant on average the reported parameters in 2010 have been calculated over 24 ± 4 steps and for 2011 measurement over 26 ± 5 steps.

### 3.1. Descriptive Statistics of the Clearance Parameters

The ANOVA on 2010 data (aged 66 to 71) and 2011 data (aged 73 to 77) showed that the gender main effect was significant for all clearance parameters (*p* < 0.001) except for minTC *(p* > 0.05*)*. Moreover, in 2011 data the age main effect was also significant for MaxHC, MaxTC1 and V_MinTC_ (*p* < 0.05).

Tables 1 and 2 show the typical range of the average of the clearance parameters for 2011 and 2010 participants, respectively. Except for the MinTC that is not different between male and female participants (*p* > 0.05), all other clearance parameters had significantly larger medians in men (*p* < 0.001) in both datasets. An interesting observation is the magnitude of V_MinTC_ that is approximately three times larger than average gait velocity in our measurements for both women and men as previously reported by Winter [13].

**Figure 5.**
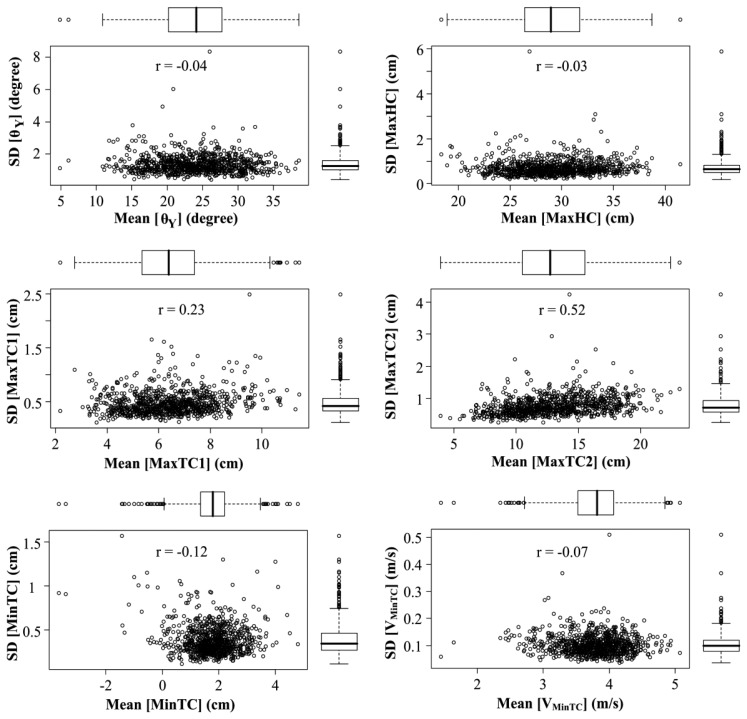
Standard deviation (SD) *vs.* mean value of the clearance parameters in 2011 measurement. The boxplots have been used to show the samples that are beyond the quartiles by one and a half interquartile range.

The clearance parameter's SD has been plotted against clearance parameter's mean value for different individuals in Figure 5 in order to investigate the dependence of parameter variations on the parameter range for 2011 measurements. The boxplot with whiskers were used to delineate the outliers. Then the correlation between the SD and mean value of the corresponding parameter has been calculated. Therefrom, a weak correlation in case of MaxTC1 (*r* = 0.23) and a moderate correlation in case of MaxTC2 (*r* = 0.52) were observed.

MaxTC1 take place early in the swing phase (20%–25%) after toe-off. Moosabhoy and Gard in [22], suggested that ankle dorsiflexion influence the foot-ground clearance at toe-off that presumably can be reflected by MaxTC1. MaxTC2 that occurs during the swing phase is considered as a control parameter for obstacle clearance [23,24]. Therefore, the study of variability of MaxTC1 and MaxTC2 can reveal the strategies used by older adults for the toe-off control and obstacle negotiation. Indeed, we observed a significant correlation between MaxTC2 and MaxHC (*r* = 0.68). This can be presumably a part of foot trajectory planning where a larger MaxHC is required to achieve larger MaxTC2 for obstacle negotiation. In [25], the authors observed a strong correlation between MinTC and MaxTC1 in 11 older subjects during treadmill walking. They suggested that intervention designed to increase MaxTC1 may also increase MinTC. However, we did not observe any significant correlation between MinTC and MaxTC1 in either of our 2010 or 2011 studies.

It worth mentioning that, the estimation of foot clearance is based on the estimation of heel and toe position from the sensor movement on foot. The model assumes a rigid shoe and a fixed heel (respectively toe) contact point at heel-strike (respectively toe-off). Considering that the average of MinTC is close to zero, a high deformation of the shoe and the change of contact point from one cycle to another can explain errors in foot clearance estimation (negative values in Figure 5). The system validation against optical motion capture in [6] showed an overall precision of 9 mm.

### 3.2. Descriptive Statistics of the Temporal and Spatial Parameters

As for 2010 data the gender main effect was significant for all measured spatiotemporal parameters (*p* < 0.001) except for $\Delta T_{ff}^{k}$ *(p* > *0*.05*)*. Even when normalized to height, the gender effect was reflected in spatial parameters (*p* < 0.05). The age main effect was significant for $\Delta T_{\text{Cyc}}^{k}$, $\Delta T_{ff}^{k}$, $\Delta T_{\text{Push}}^{k}$, Cadence, SL, SV (*p* < 0.01). Regarding 2011 data the gender main effect was significant for all measured parameters (*p* < 0.01). The age main effect was significant for $\Delta T_{St}^{k}$, SL, SV (*p* < 0.01). Moreover, gender × age interaction was statistically significant on $\Delta T_{\text{Cyc}}^{k}$, Cadence, SL, SW, SV *(p* < 0.05*)*. When normalized to height, however, the gender difference in SV mean was negated (*p* > 0.05).

Tables 3 and 4 show the range of the average of the temporal parameters for 2011 and 2010 participants, respectively. In both studies, the duration of the push phase (Δ*T_push_*) was not significantly different between men and women. The female participants were characterized by shorter gait cycle and shorter relative loading period while they had longer relative foot-flat and relative stance and higher cadence. The importance of extracting the temporal gait parameters might have been overlooked. However, in [26], the monitoring of gait parameters in 427 older adults over 5 years follow-up, showed that the increase in cadence, swing time and stance time is associated with memory decline.

Tables 5 and 6 represent the range of the average of the spatial parameters for 2011 and 2010 participants, respectively. SW (an indicator of the lateral balance) was not different between the male and female participants. In both studies, men present a faster gait velocity and longer SL and PL.

Two remarks before referring to the provided range of parameters should be considered. The parameters present the data on able-bodied older persons. However, the reported range of parameters does not necessarily represent the older adult in whom the absence of specific orthopedic condition is assured. Besides, the dataset presents the spatiotemporal and clearance parameters at a self-selected pace that should not be generalized to maximum walking velocity.

### 3.3. Inter-Relation of the Gait Speed and Clearance Parameters

Gait speed is a valuable source of information in older adults that reflects their physical performance e.g., it has been suggested that it is a predictor of functional dependence [27] and survival [28]. A decrease of 10 cm/s in gait speed has been suggested to be correlated to higher risk of fall in old age [29]. Table 7 shows the Pearson correlation coefficient between the trial average gait speed and clearance parameters for 2010 and 2011 measurements. Aside from the foot velocity at MinTC (V_MinTC_) that is strongly correlated to the gait speed, weak association between the clearance parameters and gait speed can be noticed. This observation indicates the importance of measuring the clearance parameters as independent predictors of fall risk.

## 4. Conclusions/Outlook

We presented the normative dataset of the gait parameters in community-living older persons. The assessment of the gait parameters was achieved by using previously validated shoe-worn system. This unique dataset of more than 1,400 participants is a rich source for researchers and clinicians who need a reference on the range of the temporal, spatial and clearance parameters in older individuals. The inclusion of clearance parameters allows characterizing the risky gait patterns that may lead to fall.

The dataset is also a valuable baseline for prospective cohort studies. The gait variability change and the recorded number of falls in the follow-up study helps to more precisely examine the dynamics that trigger the fall in older population. The shoe-worn system also can be used for another worthwhile line of study to investigate the obstacle negotiation strategies in older adults based on measuring the clearance parameters. The functional interpretation of the results will be the topic of another study that focuses on clinical significance of the presented parameters.

## Figures and Tables

**Table 1.** Range of the clearance parameters for 2011 measurements for men, women and total (aged 73 to 77). The parameters are heel strike pitch angle (*θ_Y_*), maximum heel clearance (MaxHC), the first and second local maxima of the toe clearance (MaxTC1 and MaxTC2 respectively), minimum toe clearance (MinTC) and minimum of the toe clearance velocity (V_MinTC_). The mean, standard deviation (SD), median ([10th;90th] percentiles) over the individual mean values have been presented. * shows the existence of significant difference between male and female participants.

**Parameter (unit)**	**Male Participant (366)**			**Female Participant (513)**			**Total (879)**		
		
	**Mean**	**SD**	**Median**	**Mean**	**SD**	**Median**	**Mean**	**SD**	**Median**
*θ_Y_* (degree)	27.5	4.5	27.8 [21.8;32.6]	21.4	4.3	21.5 [16.0;26.8]	23.9	5.3	24.0 * [16.8;31.0]
MaxHC (m)	0.32	0.03	0.32 [0.28;0.35]	0.27	0.03	0.27 [0.23;0.30]	0.29	0.01	0.29 * [0.24;0.34]
MaxTC1 (m)	0.07	0.02	0.07 [0.05;0.09]	0.06	0.01	0.06 [0.04;0.07]	0.06	0.01	0.06 * [0.04;0.08]
MaxTC2 (m)	0.16	0.03	0.16 [0.12;0.19]	0.11	0.02	0.11 [0.08;0.14]	0.13	0.03	0.13 * [0.09;0.18]
MinTC (m)	0.02	0.01	0.02 [0.01;0.03]	0.02	0.01	0.02 [0.01;0.03]	0.02	0.01	0.02 [0.01;0.03]
V_MinTC_ (m/s)	3.90	0.43	3.95 [3.38;4.35]	3.68	0.43	3.72 [3.10;4.20]	3.77	0.44	3.81 [3.20;4.28]

**Table 2.** Range of the clearance parameters for 2010 measurements for men, women and total (aged 66 to 71). The parameters are heel strike pitch angle (*θ_Y_*), maximum heel clearance (MaxHC), the first and second local maxima of the toe clearance (MaxTC1 and MaxTC2 respectively), minimum toe clearance (MinTC) and minimum of the toe clearance velocity (V_MinTC_). The mean, standard deviation (SD), median ([10th; 90th] percentiles) over the individual mean values have been presented. * shows the existence of significant difference between male and female participants.

**Parameter (unit)**	**Male Participant (239)**			**Female Participant (315)**			**Total (554)**		
		
	**Mean**	**SD**	**Median**	**Mean**	**SD**	**Median**	**Mean**	**SD**	**Median**
*θ_Y_* (degree)	27.7	4.6	27.9 [21.4;33.5]	21.9	4.4	21.8 [16.3;27.8]	24.4	5.3	24.3 * [17.9;31.2]
MaxHC (m)	0.32	0.03	0.33 [0.28;0.37]	0.27	0.03	0.28 [0.24;0.31]	0.30	0.04	0.29 * [0.25;0.35]
MaxTC1 (m)	0.08	0.02	0.07 [0.06;0.10]	0.06	0.01	0.06 [0.05;0.08]	0.07	0.02	0.07 * [0.05;0.09]
MaxTC2 (m)	0.16	0.03	0.16 [0.13;0.19]	0.11	0.02	0.11 [0.08;0.14]	0.13	0.03	0.13 * [0.09;0.18]
MinTC (m)	0.02	0.01	0.02 [0.00;0.03]	0.02	0.01	0.02 [0.01;0.03]	0.02	0.01	0.02 [0.01;0.03]
V_MinTC_ (m/s)	3.95	0.40	4.00 [3.42;4.42]	3.85	0.41	3.88 [3.31;4.33]	3.90	0.40	3.94 * [3.36;4.37]

**Table 3.** Range of the temporal parameters for 2011 measurements for men, women and total (aged 73 to 77). The parameters are gait cycle time (Δ*T_Cyc_*), relative stance duration (Δ*T_St_*), relative stance duration (Δ*T_St_*), relative load duration (Δ*T_Load_*), relative foot flat duration (Δ*T_ff_*), relative push duration (Δ*T_Push_*), and cadence. The mean, standard deviation (SD), median ([10th;90th] percentiles) over the individual mean values have been presented. * shows the existence of significant difference between male and female participants.

**Parameter (unit)**	**Male Participant (366)**			**Female Participant (513)**			**Total (879)**		
		
	**Mean**	**SD**	**Median**	**Mean**	**SD**	**Median**	**Mean**	**SD**	**Median**
Δ*T_Cyc_* (s)	1.08	0.07	1.07 [0.99;1.18]	1.05	0.09	1.04 [0.95;1.16]	1.06	0.08	1.05 * [0.96;1.17]
Δ*T_St_* (%Cycle)	61.4	1.6	61.3 [59.5;63.6]	62.2	1.9	62.0 [60.1;64.4]	61.9	1.8	61.7 * [59.7;64.0]
Δ*T_Load_* (%Stance)	14.2	3.0	14.2 [10.3;17.9]	12.1	2.7	11.7 [8.9;15.7]	12.9	3.0	12.7 * [9.2;17.0]
Δ*T_ff_* (%Stance)	59.8	5.2	59.8 [53.8;66.0]	61.3	5.7	61.6 [54.2;68.1]	60.7	5.6	61.0 * [53.9;67.5]
Δ*T_Push_* (%Stance)	26.0	3.7	25.9 [21.6;30.6]	26.6	4.3	26.3 [21.6;31.9]	26.4	4.1	26.1 [21.6;31.5]
Cadence (Step/min)	111.4	7.5	111.5 [102.2;120.6]	115.2	9.2	115.4 [103.8;126.5]	113.7	8.7	113.9 * [102.8;124.4]

**Table 4.** Range of the temporal parameters for 2010 measurements for men, women and total (aged 66 to 71). The parameters are gait cycle time (Δ*T_Cyc_*), relative stance duration (Δ*T_St_*), relative stance duration (Δ*T_St_*), relative load duration (Δ*T_Load_*), relative foot flat duration (Δ*T_ff_*), relative push duration (Δ*T_Push_*), and cadence. The mean, standard deviation (SD), median ([10th;90th] percentiles) over the individual mean values have been presented. * shows the existence of significant difference between male and female participants.

**Parameter (unit)**	**Male Participant (239)**			**Female Participant (315)**			**Total (554)**		
		
	**Mean**	**SD**	**Median**	**Mean**	**SD**	**Median**	**Mean**	**SD**	**Median**
Δ*T_Cyc_* (s)	1.09	0.08	1.08 [0.99;1.21]	1.04	0.08	1.03 [0.95;1.14]	1.06	0.09	1.06 * [0.96;1.17]
Δ*T_St_* (%Cycle)	61.2	1.6	61.3 [59.2;63.3]	61.9	1.8	61.7 [59.8;64.2]	61.6	1.8	61.6 * [59.4;63.8]
Δ*T_Load_* (%Stance)	14.5	3.0	14.1 [10.9;18.6]	12.3	2.9	11.8 [9.0;15.9]	13.2	3.1	13.1 * [9.3;17.4]
Δ*T_ff_* (%Stance)	59.8	5.4	59.8 [53.1;66.4]	60.6	5.7	60.8 [53.3;67.8]	60.3	5.6	60.3 * [53.2;67.1]
Δ*T_push_* (%Stance)	25.7	4.2	25.5 [20.8;30.6]	27.1	4.3	27.1 [22.1;32.4]	26.5	4.3	26.3 [21.5;31.9]
Cadence (Step/min)	110.4	8.2	110.6 [99.0;120.6]	115.6	8.7	115.9 [105.1;126.1]	113.3	8.9	113.6 * [102.2;124.6]

**Table 5.** Range of the spatial parameters for 2011 measurements for men, women and total (aged 73 to 77). The parameters are stride velocity (SV), stride length (SL), stride width (SW) and path length (PL). The mean, standard deviation (SD), median ([10th;90th] percentiles) over the individual mean values have been presented. * shows the existence of significant difference between male and female participants.

**Parameter (unit)**	**Male Participant (366)**			**Female Participant (513)**			**Total (879)**		
		
	**Mean**	**SD**	**Median**	**Mean**	**SD**	**Median**	**Mean**	**SD**	**Median**
SV (m/s)	1.26	0.17	1.27 [1.04;1.46]	1.15	0.17	1.16 [0.92;1.35]	1.19	0.18	1.20 * [0.95;1.41]
SL (m)	1.34	0.15	1.35 [1.16;1.52]	1.18	0.13	1.19 [1.02;1.35]	1.25	0.16	1.25 * [1.05;1.45]
SW (m)	0.04	0.01	0.04 [0.02;0.06]	0.04	0.01	0.04 [0.02;0.06]	0.04	0.01	0.04 [0.02;0.06]
PL (% SL)	104.7	0.6	104.6 [104.0;105.5]	104.3	0.7	104.2 [103.5;105.2]	104.5	0.7	104.4 * [103.6;105.4]

**Table 6.** Range of the spatial parameters for 2010 measurements for men, women and total (aged 66 to 71). The parameters are stride velocity (SV), stride length (SL), stride width (SW) and path length (PL). The mean, standard deviation (SD), median ([10th;90th] percentiles) over the individual mean values have been presented. * shows the existence of significant difference between male and female participants.

**Parameter (unit)**	**Male Participant (239)**			**Female Participant (315)**			**Total (554)**		
		
	**Mean**	**SD**	**Median**	**Mean**	**SD**	**Median**	**Mean**	**SD**	**Median**
SV (m/s)	1.27	0.16	1.30 [1.06;1.47]	1.22	0.17	1.23 [0.99;1.42]	1.24	0.17	1.26 * [1.02;1.45]
SL (m)	1.38	0.13	1.39 [1.20;1.53]	1.25	0.13	1.27 [1.09;1.41]	1.30	0.14	1.31 * [1.12;1.48]
SW (m)	0.04	0.01	0.04 [0.02;0.06]	0.04	0.01	0.04 [0.03;0.06]	0.04	0.01	0.04 [0.02;0.06]
PL (% SL)	104.9	1.5	104.6 [103.9;105.9]	104.4	1.0	104.3 [103.5;105.2]	104.6	1.3	104.4 * [103.6;105.6]

**Table 7.** Correlation values between clearance parameters and gait speed for male (M), female (F) and all participants (All). For all of the reported correlations, *p* < 0.001.

	**Database**	***θ****_Y_*	**MaxHC**	**MaxTC1**	**MaxTC2**	**MinTC**	**V_MinTc_**
***Correlation with Gait Speed***	2010	M = 0.46	M = 0.16	M = 0.15	M = 0.40	M = 0.21	M = 0.94
		F = 0.51	F = 0.27	F = 0.09	F = 0.47	F = 0.10	F = 0.95
		**All = 0.50**	**All = 0.28**	**All = 0.03**	**All = 0.43**	**All = 0.16**	**All = 0.95**

	2011	M = 0.58	M = 0.46	M = 0.35	M = 0.55	M = 0.23	M = 0.86
		F = 0.38	F = 0.39	F = 0.14	F = 0.23	F = 0.12	F = 0.62
		**All = 0.49**	**All = 0.42**	**All = 0.25**	**All = 0.40**	**All = 0.18**	**All = 0.75**
